# Impact of Pharmacogenetics on High-Dose Methotrexate Toxicity in Pediatric Oncology

**DOI:** 10.3390/pharmaceutics17050585

**Published:** 2025-04-29

**Authors:** Luciana Maria Marangoni-Iglecias, Almudena Sánchez-Martin, Laura Elena Pineda-Lancheros, Yasmín Cura, Noelia Marquez-Pete, José María Gálvez-Navas, Nerea Báez-Gutiérrez, Adrián Manuel de La Jara-Vera, Emilia Urrutia-Maldonado, Cristina Pérez-Ramírez, Alberto Jiménez-Morales

**Affiliations:** 1 Clinical Analysis Laboratory Unit, Hospital Universitário Maria Aparecida Pedrossian HUMAP-UFMS. Av. Sen. Filinto Müler, 355-Vila Ipiranga, Campo Grande 79080-190, Brazil; 2 Pharmacy Service, Pharmacogenetics Unit, University Hospital Virgen de las Nieves, Avda. de las Fuerzas Armadas 2, 18004 Granada, Spain; almudena.sanchez.martin.sspa@juntadeandalucia.es (A.S.-M.); yasmin.cura@gmail.com (Y.C.); noeliamarquezpete@gmail.com (N.M.-P.); adriandljv@correo.ugr.es (A.M.d.L.J.-V.); perezramirez87@ugr.es (C.P.-R.); alberto.jimenez.morales.sspa@juntadeandalucia.es (A.J.-M.); 3 Pharmacogenetics Unit, Pharmacy Service, Virgen de las Nieves University Hospital, 18014 Granada, Spain; lepinedal@unal.edu.co; 4 Instituto de Investigación Biosanitaria ibs.GRANADA, Avda. de Madrid 15, 18012 Granada, Spain; 5 Consorcio de Investigación Biomédica en Red de Epidemiología y Salud Pública (CIBERESP), 28029 Madrid, Spain; jmaria.galvez.easp@juntadeandalucia.es; 6 Cancer Registry of Granada, Andalusian School of Public Health, Cuesta del Observatorio 4, 18011 Granada, Spain; 7 Department of Biochemistry and Molecular Biology II, Faculty of Pharmacy, Campus Universitario de Cartuja, University of Granada, 18011 Granada, Spain; 8 Pharmacy Service Hospital Virgen del Rocío, Avenida De Manuel Siurot S/n, 41013 Seville, Spain; nerea.baez.sspa@juntadeandalucia.es; 9 Assistant Physician, University Hospital Virgen de las Nieves, Avda. de las Fuerzas Armadas 2, 18004 Granada, Spain; emilia.urrutia@gmail.com

**Keywords:** HDMTX, genetic polymorphisms, childhood cancer, pharmacogenetics

## Abstract

**Background**: Childhood cancers represent a heterogeneous group of malignancies and remain one of the leading causes of mortality among children under 14 years of age, ranking second only to accidental injuries, and fourth among individuals aged 15 to 19 years. Despite notable improvements in cure rates, a substantial proportion of patients experience acute or long-term toxicities associated with treatment. Methotrexate (MTX), a chemotherapeutic agent, has been employed effectively for over six decades in the management of pediatric malignancies. High-dose methotrexate constitutes a cornerstone of pediatric cancer therapy; however, its clinical utility is frequently constrained by dose-limiting toxicities. **Objectives:** This study investigates the impact of genetic polymorphisms in genes involved in nucleotide metabolism, as well as methotrexate and folate metabolic pathways, on treatment-related toxicity in childhood cancer. **Methods:** Using real-time polymerase chain reaction, 14 polymorphisms across 12 genes were analyzed in a cohort of 107 patients. Toxicity was assessed according to the Common Terminology Criteria for Adverse Events v. 5.0. **Results:** Multivariate logistic regression analysis revealed that the male sex (*p* = 0.3) and the AA genotype of *MTHFD1* rs2236225 were associated with grade III–IV gastrointestinal toxicity (*p* = 0.03), while the A allele of *MTHFR* rs1801133 and the AA genotype of *GSTP1* rs1695 were associated with grade I–IV hematologic toxicity (*p* < 0.01 and *p* = 0.02, respectively). **Conclusions:** High-dose methotrexate (HDMTX) is a critical agent in the treatment of childhood cancers. Our findings suggest that genetic polymorphisms within methotrexate and folate metabolic pathways may serve as potential predictive biomarkers of treatment-related toxicity.

## 1. Introduction

Childhood cancers constitute a heterogeneous group of malignancies that vary in terms of their etiology, incidence, survival rates, and the risk of late sequelae [1,2]. According to the American Cancer Society, approximately 14,910 new cases of cancer were diagnosed in children and adolescents in the United States in 2024, with an estimated 1590 deaths. The most frequently diagnosed types of childhood cancer are acute lymphoblastic leukemia (ALL), followed by lymphomas and neoplasms of the nervous system. Cancer remains one of the leading causes of death in children under 14 years old, ranking second after accidents, and fourth in individuals aged 15 to 19 years. Since 2016, the overall incidence rate for invasive cancer in children has stabilized. However, leukemia and lymphoma rates are slowly increasing among adolescents, resulting in a 1% annual rise in overall cancer incidence within this age group from 2015 to 2019. Cancer mortality has steadily declined in both children and adolescents, with reductions of 70% and 63%, respectively [3,4]. Despite achieving high cure rates, a significant proportion of patients experience acute or long-term toxicity associated with childhood cancer treatment [5,6]. Specifically, 1–3% of patients diagnosed with ALL may eventually die due to the toxic effects of treatment [7]. Furthermore, the number of individuals surviving childhood cancer continues to increase. This increased life expectancy has led to growing attention and concern regarding the potential long-term consequences of cancer treatments administered early in life [1].

**Figure 1:**
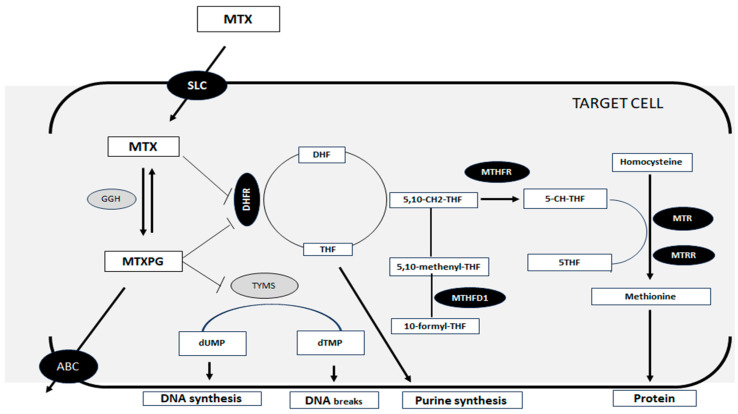
Simplified scheme representative of the intracellular MTX pathway. MTX: methotrexate; SLC: solute carrier organic anion transporter; MTXPG: methotrexate polyglutamated form; GGH: γ-glutamyl hydrolase; DHFR: dihydrofolate reductase; DHF: dihydrofolate; TYMS: thymidylate synthetase; dUMP: deoxyuridine monophosphate; dTMP: deoxythymidine monophosphate; THF: tetrahydrofolate; 5,10-CH2-THF: 5,10-methylenetetrahydrofolate; MTFR: methylenetetrahydrofolate reductase; MTHFD1: methylenetetrahydrofolate dehydrogenase; 5-CH-THF: 5-methyltetrahydrofolate; MTR: methylenetetrahydrofolate-homocysteine methyltransferase; MTRR: 5-methylenetetrahydrofolate-homocysteine methyltransferase reductase. Figure produced by the author.

Methotrexate (MTX) is a chemotherapeutic agent that has been effectively used for more than 60 years in the treatment of childhood cancer [8,9]. MTX acts as a folic acid analog in cell metabolism, and it is used in high doses to treat various types of malignant neoplasms [5,6]. Solute carrier family (SLC) transporters facilitate MTX entry into cells, while ATP-binding cassette (ABC) transporters mediate its efflux [6,10]. Once inside the cell, MTX undergoes polyglutamylation (MTXPG), which increases its affinity for target enzymes and enhances its intracellular retention. MTX binds to the enzyme dihydrofolate reductase (DHFR), disrupting the production of tetrahydrofolate, a crucial cofactor for enzymes involved in DNA, RNA, and protein synthesis. Additionally, MTX metabolites inhibit enzymes involved in de novo purine synthesis, thereby impairing DNA replication and cell proliferation [5,10,11,12]. MTXPG can be actively transported into lysosomes, wherein it undergoes hydrolysis, facilitating the removal of the drug and short-chained polyglutamates from the cell [10,13,14]. Furthermore, the depletion of tetrahydrofolate disrupts the complex metabolism of homocysteine remethylation to methionine, contributing to cell death. This process involves several other enzymes, including methylenetetrahydrofolate reductase (MTHFR) and methionine synthase (MTR) (Figure 1). Additionally, proteins involved in nucleotide metabolism may also influence MTX toxicity [15].

High-dose MTX (HDMTX) (>500 mg/m^2^) [16] is extensively used in treatment protocols for childhood cancers, including leukemias, lymphomas, and osteosarcoma (OS) [5,17]. Despite its efficacy, its lack of specificity often causes collateral damage in healthy tissues, particularly in rapidly dividing cells of the bone marrow, mucous membranes, liver, and kidneys [16,17]. This leads to significant toxicity, manifesting as gastrointestinal, hepatic, and hematologic complications, which can compromise treatment continuity and outcomes [18,19,20].

Gastrointestinal toxicity commonly manifests nausea (30–60%), vomiting (13–33%), and mucositis (76%), despite the application of prophylactic measures. Consequences, such as dehydration, weight loss, and treatment delays, are frequent [17,21,22]. Hematologic toxicity, characterized by anemia and leukopenia, is attributed to the impact of MTX on bone marrow cells. In severe cases, it may result in pancytopenia, bleeding, and an increased risk of infection [12,23,24]. To mitigate these risks, close monitoring of serum MTX levels, adequate hydration, urine alkalinization, and timely leucovorin rescue are all essential strategies. Regular blood counts also facilitate the early detection of hematologic toxicity, allowing for prompt interventions, including dose adjustments, treatment delays, or discontinuation, if necessary [25].

The type and severity of toxicity vary among patients, partly due to genetic factors. Pharmacogenetics offers the potential to predict MTX toxicity by identifying genetic polymorphisms that affect treatment outcomes [16,26,27]. Several studies have examined the potential association between polymorphisms in genes involved in MTX and folate pathways and the development of toxicity in childhood cancer and other diseases [8,25,28]. However, this relationship remains unclear. Many studies on candidate genes have produced inconsistent results, frequently lacking the replication and validation necessary to confirm the findings [19,29,30]. This presents a significant challenge in developing clinical guidelines and integrating MTX pharmacogenetics into routine medical practice.

Based on the available evidence, our study aimed to investigate the influence of single nucleotide polymorphisms (SNPs) in MTX, folate, and nucleotide pathway genes on antineoplastic therapy toxicity. We examined 107 children diagnosed with cancer who were undergoing HDMTX treatment retrospectively, analyzing the toxicities documented in their medical records. A total of 14 SNPs in genes involved in MTX pharmacokinetics and pharmacodynamics were analyzed, including methionine synthase reductase (*MTRR)* rs1801394, *MTHFR* rs1801133, *MTHFR* rs1801131, methylenetetrahydrofolate dehydrogenase, cyclohydrolase, and formyltetrahydrofolate synthetase 1 *MTHFD1* rs2236225, *MTR* rs180508, *DHFR* rs70991108, ATP-binding cassette subfamily B member 1 (ABCB1) rs1128503, ATP-binding cassette, subfamily C, member 1 (*ABCC1*) rs246240, ATP-binding cassette, subfamily C, member 2 (*ABCC2*) rs717620, solute carrier organic anion transporter family member 1B1 (*SLCO1B1*) rs11045879, *SLCO1B1* rs4149081, adenosine A2a receptor (*ADORA2A*) rs5760410, inosine triphosphatase (*ITPA*) rs7270101, and glutathione S-transferase pi 1 (*GSTP1* rs1695).

## 2. Materials and Methods

### 2.1. Study Design

A retrospective cohort study was conducted.

### 2.2. Study Population

The present study included 107 patients aged 1–17 years, of Caucasian origin, diagnosed with childhood cancer, and undergoing HDMTX treatment. The patients were treated at the Hospital Virgen de las Nieves in Granada, Spain, and Virgen del Rocío in Seville, Spain, between November 2005 and November 2021. Patients with unavailable or incomplete medical records for follow-up, those who had not completed the HDMTX treatment cycle, and carriers of mutated polymorphisms for thiopurine S-methyltransferase (*TPMT*) and Nudix hydrolase 15 (*NUDT15*), as described in the Clinical Pharmacogenetics Implementation Consortium (CPIC) guidelines for thiopurines, were excluded from the study because polymorphisms in these genes that result in reduced enzymatic function are associated with myelosuppression [31]. No patients were administered concomitant medication with the same toxicity profile as MTX.

### 2.3. Ethics Statements

The study was approved by the Ethics and Research Committee of Granada (code: 0388-M1-19) and conducted following the Declaration of Helsinki. The patients who participated in the study or their legal representatives provided informed consent for the collection and genetic analysis of their saliva samples and for their donation to the Andalusian Public Health System Biobank. Alphanumeric codes were used to identify the samples.

### 2.4. Sociodemographic and Clinical Variables

Sociodemographic and clinical data were collected from the patients’ medical records. The data included sex, family history of cancer, cancer diagnosis, age at diagnosis, and concomitantly administered antineoplastic drugs.

Data on all the toxicities documented in the medical records that occurred within four weeks of the last HDMTX cycle or the commencement of the subsequent phase of treatment, whichever occurred first, were collected. These toxicities included gastrointestinal toxicity (nausea and vomiting, diarrhea, mucositis), hematologic toxicity (neutropenia, thrombocytopenia, anemia, pancytopenia, febrile neutropenia), hepatotoxicity, pulmonary toxicity (pulmonary viral or bacterial infection), and renal toxicity. The toxicities were classified as present or absent and were graded according to the National Cancer Institute Common Toxicity Criteria, version 5.0 [32]. The toxicity grades were categorized into grades I–II for mild and III–IV for severe toxicities. In the event that a patient experienced multiple episodes of the same toxicity, the highest grade was applied.

### 2.5. Genetic Variables

#### 2.5.1. DNA Isolation

Saliva samples were collected using an oral swab, and DNA was extracted using the QIAamp DNA Mini Kit (Qiagen GmbH, Hilden, Germany), following the manufacturer’s protocol for purifying DNA. The extracted DNA was then stored at −40 °C. The DNA quantification and purity were assessed using a NanoDrop 2000 UV spectrophotometer, measuring the 260/280 and 260/230 absorbance ratios (NanoDrop Technologies Inc., Wilmington, DE, USA).

#### 2.5.2. Detection of Genetic Polymorphisms

A total of 14 SNPs with a minor allele frequency greater than 15% in the Spanish population [33] were selected based on their relevance to the pharmacokinetics and pharmacodynamics of methotrexate (MTX), as well as their established or potential associations with MTX-related toxicity [34]. The analyzed polymorphisms included *MTRR* rs1801394, *MTR* rs1805087, *MTHFR* rs1801133 and rs1801131, *MTHFD1* rs2236225, *GSTP1* rs1695, *ABCB1* rs1128503, *ABCC1* rs246240, *ABCC2* rs717620, and the insertion/deletion polymorphism *DHFR* rs7099110. Additionally, polymorphisms involved in nucleotide metabolism, specifically *ADORA2A* rs5760410 and *ITPA* rs7270101, were included, as both have been previously implicated in MTX toxicity. Furthermore, the *SLCO1B1* polymorphisms, rs11045879 and rs4149081, were evaluated in accordance with the recommendations by the French National Network of Pharmacogenetics (RNPGx) [35].

The genetic polymorphisms were determined via a real-time polymerase chain reaction (PCR) allelic discrimination assay, using TaqMan™ probes (ABI Applied Biosystems, QuantStudio 3 Real-Time PCR System, 96 wells), according to the manufacturer’s instructions (Table 1). This procedure is based on the use of fluorescent oligonucleotide probes, labeled with a fluorescent reporter and a quencher; the two are tightly coupled if the probe does not hybridize to its target sequence, thus there is no amplification and no fluorescence signal. When it hybridizes, conformational changes occur in the reporter and quencher, allowing the 5′–3′ exonuclease activity of Taq polymerase to break this bond, allowing the fluorescence emitted by the reporter to be released and captured by the device. Each allele is labeled with a different fluorochrome, so that the genotype is determined according to the fluorescence captured by the device [36]. The criterion for the quality control of the SNPs was a *p*-value > 0.05 in the Hardy–Weinberg equilibrium (HWE) test.

#### 2.5.3. Statistical Analysis

Qualitative variables were expressed as frequencies and percentages, while quantitative variables were expressed as medians and percentiles (p25–p75). Normality was assessed using the Kolmogorov–Smirnov test. In the bivariate analysis, Fisher’s exact test and χ^2^ tests were used for qualitative variables, while the Mann–Whitney test was applied to non-normally distributed quantitative variables. SNP association models were defined as follows: dominant (DD, Dd) vs. dd, recessive DD vs. (Dd, dd), and genotypic DD vs. dd and Dd vs. dd. Univariate logistic regression analysis was performed to evaluate significant associations and calculate the adjusted odds ratio (OR) with a 95% confidence interval (CI) for potential prognostic factors of toxicity. Significant associations from the bivariate analysis underwent multivariate logistic regression, using the backward stepwise method. The HWE model and MAF were assessed. The linkage disequilibrium (LD) was determined by calculating Lewontin’s D prime coefficient and the LD coefficient (R^2^). An LD chart was generated using the Haploview v. 4.2 software. All the tests were two sided, with a significance level set at *p* < 0.05, and were conducted using the software PLINK v. 1.9, for genome-wide association analysis [37], and R v. 4.2.0 [38].

## 3. Results

### 3.1. Sociodemographic and Clinical Variables

The study cohort comprised a total of 107 Caucasians patients, 49 (45.79) women and 58 (54.21) men. The patients were aged between 1–17 years at the time of cancer diagnosis. The median age at diagnosis was 6 years (range 4–8 years) for women and 6.9 (3–11.5) for men. Regarding diagnosis, among the women, 65.31% (32/49) had acute lymphoblastic leukemia (ALL), 14.29% (7/49) had non-Hodgkin’s lymphoma (NHL), 14.29% (7/49) had osteosarcoma (OS), and 6.12% (3/49) had ependymoma (EPN). Among the male patients, regarding diagnosis, 58.62% (34/58) had ALL, 20.69% (12/58) had NHL, 17.24% (10/58) had OS, 1.72% (1/58) had EPN, and 1.72% (1/58) had undifferentiated sarcoma of small cells. Most patients, 75.51% (37/49) women and 77.59% (45/58) men, received the concomitant administration of other antineoplastic drugs, in addition to HDMTX.

In the cohort examined, 73.43% (36/49) of women and 62.07% (36/58) of men exhibited delayed elimination of HDMTX, 67.35% (33/49) of women and 86.21% (50/58) experienced toxicity graded I–IV, and 61.22% (30/49) of women and 72.41% (42/58) of men encountered severe toxicity. The most common reported toxicities in patients were hematologic, affecting 53.06% (26/49) of women and 68.97% (40/58) of men, and gastrointestinal toxicity, impacting 53.6% (26/49) of women and 67.24% (40/58) of men, followed by liver toxicity. Moreover, 36.73% (18/49) of women and 34.48% (20/589) of men experienced toxicity, with renal toxicity affecting 4.08% (2/49) of women and 8.62% (5/58) of men, cutaneous toxicity affecting 2.04% (1/49) of women and 12.07% (8/58) of men, and pulmonary toxicity affecting 8.16% (4/49) of women and 6.90% (4/58) of men.

Hematological toxicity most frequently included isolated neutropenia, in 51.02% of women and 67.24% of men, and thrombocytopenia, in 24.49% of women and 46.55% of men. Nausea and vomiting were the most common GI toxicities, reported by 40.82% of women and 43.10% of men, followed by mucositis (14.29% of women and 32.76% of men) and diarrhea (12.24% of women and 15.52% of men). The complete sociodemographic data are presented in Table 1.

### 3.2. Sociodemographic and Clinical Variables Associated with HDMTX Toxicity

The male sex was associated with an increased likelihood of experiencing general toxicity grade I–IV (*p* = 0.02; OR = 2.30; 95%CI = 1.06–9.07), as well as severe gastrointestinal toxicity (*p* = 0.016; OR = 3.87; 95%CI = 1.11–17.32) and mucositis (grades I–IV) (*p* = 0.026; OR = 2.89; 95%CI = 1.02–9.07). Additionally, males exhibited an elevated risk of developing thrombocytopenia (grades I–IV) (*p* = 0.02; OR = 2.66; 95%CI = 1.09–6.78). Patients diagnosed with ALL showed an increased risk of severe hepatic toxicity (*p* < 0.001; OR = 5.81; 95%CI = 1.07–108.36) and prolonged MTX clearance (>48 h) (*p* = 0.025; OR = 3.16; 95%CI = 1.11–9.34). The diagnosis of NHL was found to be correlated with severe gastrointestinal toxicity (*p* = 0.029; OR = 3.93; 95%CI = 1.20–12.85) and mucositis (grades I–IV) (*p* < 0.001; OR = 5.60; 95%CI = 1.90–17.37). The diagnosis of OS was associated with an increased susceptibility to gastrointestinal toxicity (grades I–IV) (*p* < 0.001; OR = 7.96; 95%CI = 2.03–53.13), nausea and vomiting (grades I–IV) (*p* < 0.001; OR = 10.00; 95%CI = 2.89–48.86.), severe hepatic toxicity (*p* < 0.001; OR = 3.11; 95%CI = 1.23–6.11), and prolonged MTX clearance (>48 hrs.) (*p* = 0.025; OR = 6.41; 95%CI = 1.49–35.20). The age at diagnosis was associated with gastrointestinal toxicity (grades I–IV) (*p* < 0.001; OR = 1.71; 95%CI = 1.05–1.31), specifically with nausea and vomiting (*p* < 0.001; OR = 1.20; 95%CI = 1.09–1.34.). The concurrent administration of other chemotherapeutics was associated with an elevated risk of severe hematological toxicity (*p* = 0.020; OR = 2.96; 95%CI = 1.06–8.91) (Table 2).

### 3.3. Genotype Distribution

**Figure 2:**
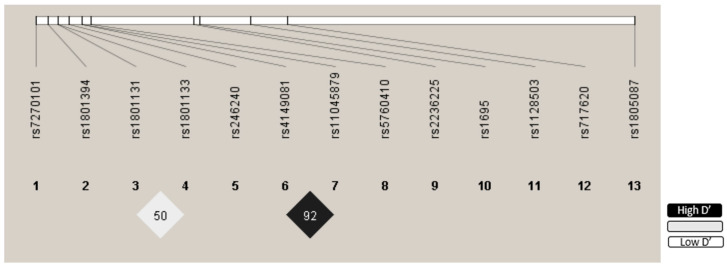
Linkage disequilibrium (LD). Polymorphisms: 1-*ITPA*; 2-*MTRR*; 3-*MTHFR*; 4-*MTHFR*; 5-*ABCC1*; 6-*SLCO1B1*; 7-*SLCO1B1*; 8-*ADORA2A*; 9-*MTHFD1*; 10-*GSPT1*; 11-*ABCC1*; 12-*ABCC2*; 13-*MTR*.

The distribution of the studied polymorphisms was consistent with the HWE model. The LD and R^2^ values are presented in Table S1. The polymorphisms, *SLCO1B1* rs4149081 and *SLCO1B1* rs11045879, were in linkage disequilibrium (R^2^ = 0.847 D’ = 0.929 (Table S2). Figure 2 depicts the LD chart. The SNPs included in the study exhibited an MAF > 15%, and none were excluded from the study (Table S3).

### 3.4. Polymorphisms Associated with Toxicity

In the bivariate analysis, the AA genotype (AA vs. G) of the *GSTP1* rs1695 polymorphism was found to be associated with severe overall toxicity (*p* = 0.03; OR = 2.55; 95% CI = 1.02–6.70) and neutropenia (grades I–IV) (*p* = 0.02; OR = 2.64; 95% CI = 1.11–6.50). The G allele (G vs. AA) of the *MTHFR* rs1801133 polymorphism was associated with severe gastrointestinal toxicity (*p* = 0.02; OR = Inf.; 95%CI = 1.14-Inf.), whereas the A allele (A vs. GG) was associated with hematologic toxicity (grades I–IV) (*p* < 0.01; OR = 3.45; 95% CI = 1.36–9.16) and, specifically, with neutropenia (grades I–IV) (*p* = 0.01; OR = 3.07; 95% CI = 1.21–8.02). The AA genotype (AA vs. G) of the *MTHFD1* rs2236225 polymorphism was found to be associated with an greater risk of severe gastrointestinal toxicity grade III–IV compared to toxicity grade I–II (*p* = 0.01; OR = 6.38; 95% CI = 1.17–45.00), as well as the G allele (G vs. AA) of the *ABCB1* rs1128503 polymorphism that showed a tendency to associate with highest toxicity grade III–IV in comparison to those of grade I–II (*p* = 0.05; OR = 6.93; IC95% = 0.90–317.32). Furthermore, the double insertion (II vs. D) of the *DHFR* rs70991108 polymorphism was associated with gastrointestinal toxicity (grades I–IV), particularly nausea and vomiting (*p* < 0.01; OR = 3.30; 95% CI = 1.38–8.11). The AA genotype (AA vs. G) of the *MTR* rs1805087 polymorphism was associated with concomitant neutropenia and thrombocytopenia (*p* = 0.01; OR = 2.69; 95% CI = 1.10–6.68) (Table S4).

The multivariate logistic regression analysis revealed that the male sex and the AA genotype (AA vs. AG/GG) of *MTHFD1* rs2236225 were associated with severe gastrointestinal toxicity (*p* = 0.03; OR = 3.71; 95% IC = 1.20–14.10 and *p* = 0.03; OR = 6.15; 95% CI = 1.29–35.41, respectively) (Table 3). Furthermore, the A allele (A vs. GG) of *MTHFR* rs1801133 (*p* < 0.01; OR = 4.23; 95% CI = 1.73–10.98) and the AA genotype (AA vs. G) of *GSTP1* rs1695 (*p* = 0.02; OR = 2.88; 95% CI = 1.23–7.17) were associated with hematologic toxicity (grades I–IV). The hematological toxicities and variables confirm their association after adjustment for FDR, while gastrointestinal toxicities do not maintain this association (Table 3). No associations were observed between the studied polymorphisms and hepatic, pulmonary, dermatologic, or renal toxicity, in the study population.

## 4. Discussion

The toxicity observed during HDMTX treatment for childhood cancer exhibits considerable inter- and intra-patient variability, even when the same dosage is administered [1,16]. This variability can be partially attributed to the influence of genetic polymorphisms that affect the activity, expression, or interaction of proteins involved in MTX metabolism, transport, and targets, thereby altering its pharmacokinetics and pharmacodynamics. The identification of biomarkers of MTX toxicity is essential to personalize antineoplastic therapy and improve its safety. Our findings revealed a significant association between be the male sex and *MTHFD1* rs2236225 SNP, with the occurrence of severe gastrointestinal toxicity, as well as significant associations between the *MTHFR* rs1801133 and *GSTP1* rs1695 SNPs and hematological toxicity (grades I–IV).

### 4.1. Sex

It is essential to consider sex-based biological differences that influence medication metabolism and toxicity in pediatric cancer treatment. Pharmacokinetic variables, including variations in hepatic clearance, renal function, and metabolic pathways, have been identified as contributing factors to high-dose methotrexate (HDMTX) toxicity. Our analysis demonstrated that the male sex was associated with a significantly increased risk of grade III–IV gastrointestinal toxicity following HDMTX administration (*p* = 0.003; OR = 3.71; 95% CI: 1.20–14.10). The current scientific literature remains limited in regard to the provision of data elucidating the impact of sex on the manifestation of toxicity during HDMTX treatment, particularly within pediatric populations. A retrospective study conducted in 2015 by Wiczer et al., involving 170 adult patients in the United States, who were treated with HDMTX across various chemotherapy regimens, supports our findings. Their study indicated that the male sex was significantly associated with an increased risk of HDMTX-related toxicity, particularly nephrotoxicity (OR = 2.13; 95% CI: 1.27–4.18; *p* = 0.006) [39]. In contrast, a study conducted in 2016 by Meeske et al. in the United States, utilizing data from the Children’s Cancer Group (CCG) high-risk acute lymphoblastic leukemia (ALL-HR) trial (CCG-1961, n = 2054, including both Hispanic and non-Hispanic patients) and the standard-risk cohort (CCG-1991, n = 3054), revealed that female patients in the high-risk ALL group experienced a higher incidence of hospitalization (*p* = 0.006), treatment delays during the consolidation phase, and more severe adverse events compared to their male counterparts. Furthermore, female patients exhibited an increased risk of treatment-related mortality, with a significantly higher mortality rate observed five months after treatment initiation (Hazard Ratio = 2.8; 95% CI: 1.5–5.3; *p* = 0.002) [40].

### 4.2. MTHFD1 rs2236225

The *MTHFD1 gene*, located on chromosome 14, encodes an enzyme that exhibits three distinct activities: 5,10-methylenetetrahydrofolate dehydrogenase, 5,10-methylenetetrahydrofolate cyclohydrolase, and 10-formyltetrahydrofolate synthetase [41,42]. The subsequent reactions catalyzed by this enzyme are critical for folate metabolism and purine biosynthesis, and are essential for DNA synthesis, methylation, and repair [20]. The *MTHFD1* rs2236225 polymorphism is defined by a guanine-to-adenine substitution at position 1958, which leads to an alanine-to-glycine change at position 653 of the 10-formyltetrahydrofolate synthetase, resulting in decreased enzymatic activity [25,43]. The evidence on the impact of this polymorphism on MTX toxicity is limited, and the available studies report conflicting results. Some studies suggest that the *MTHFD1* rs2236225 SNP may influence MTX pharmacokinetics, although the findings remain inconclusive [25]. Our study revealed an association between the AA genotype of *MTHFD1* rs2236225 (AA vs. AG/GG) and the male sex and severe gastrointestinal toxicity. To our knowledge, no previous studies have reported an association between this SNP and MTX gastrointestinal toxicity [19,44,45]. A study published in 2011 reported a significant association between an increased likelihood of hematologic toxicity (anemia) and the *MTHFD1* rs2236225 AA/AG genotypes in 50 children from multiethnic ancestry (Caucasian, Afro-Caribbean, Indian) with OS, treated with MTX (*p* = 0.044) [46]. Erčulj et al. reported that this SNP may have a protective effect against hepatic toxicity in 167 Slovenian children with ALL undergoing HDMTX treatment (*p* = 0.009) [44]. Conversely, another study conducted in Slovenia, investigated the potential association between *MTHFD1* rs2236225 and hematologic toxicity, mucositis, and hepatic toxicity in a cohort of 28 children diagnosed with NHL undergoing HDMTX treatment. However, the study found no evidence supporting this relationship [19]. These conflicting results may be due to differences in treatment protocols for these different pathologies and also the small sample size.

### 4.3. MTHFR rs1801133

The *MTHFR,* located in chromosome 1, encodes the MTHFR enzyme, which catalyzes the conversion of 5,10-methylentetrahydrofolate (5,10-CH_2_-THF) to 5-methyltetrahydrofolate (5-MTHF) [47,48]. The MTHFR enzyme is critical for folate homeostasis, purine and DNA synthesis and repair. Although it is not a direct target of MTX, MTHFR participates in metabolic pathways wherein several chemotherapeutic agents, including MTX, exert their effects [21,34,38]. The *MTHFR* rs1801133 polymorphism is characterized by a cytosine-to-thymine (C677T) substitution, leading to the replacement of alanine with valine at codon 222 [49]. This change reduced the enzymatic activity by 40% in heterozygous carriers and 70% in homozygotes [5,50]. The relationship between *MTHFR* rs1801133 and MTX-related toxicities in cancer patients has been the subject of extensive research, particularly in patients diagnosed with ALL. However, current evidence remains inconclusive [5,51,52]. This may be due to the fact that the metabolism of MTX is quite complex and involves, directly and indirectly, a large number of enzymes whose polymorphisms can influence, alone or together, the appearance of toxicities during treatment.

In our study, the multivariate logistic regression analysis revealed an association between the *MTHFR* rs1801133 A allele (A vs. GG) and hematologic toxicity (grades I–IV) (*p* < 0.01). This finding aligns with those of a study conducted in 2014 in Slovenia on a cohort of 30 European children with NHL, which reported an association between the *MTHFR* rs1801133 AA/AG genotypes (A vs. GG) and leukopenia (*p* = 0.006), as well thrombocytopenia (*p* = 0.041) [19]. Similarly, in 2015, Aráoz and collaborators reported that in a cohort of 286 Argentine children diagnosed with ALL, carriers of the *MTHFR* rs1801133 A allele (A vs. GG) had a higher risk of leukopenia and neutropenia (*p* = 0.004 y *p* = 0.010, respectively) when treated with MTX at a dose of 2 g/m^2^/day. This study found no association between the SNP and hematologic toxicity at a MTX dose of 5 g/m^2^/day, nor was any association observed between the polymorphism and thrombocytopenia at any dose [53]. Conversely, a study conducted in 2023 on 271 Chinese children undergoing ALL treatment observed an association between the *MTHFR* rs1801133 GG genotype (GG vs. A) and neutropenia (*p* < 0.05), as well as thrombocytopenia (*p* < 0.05), among patients at low risk of relapse. It is important to note that this finding was not confirmed in the multivariate analysis when the high-risk group of patients was examined [54]. Furthermore, Giletti et al. (2017) reported an association between the *MTHFR* rs1801133 GG genotype (GG vs. A) and hematologic toxicity (*p* < 0.005) in a cohort of 41 adult patients undergoing treatment for ALL and NHL in Uruguay [55].

### 4.4. GSTP1 rs1695

The *GSTP1*, located on chromosome 11, is highly polymorphic and encodes the GSTP1 enzyme, which catalyzes the conjugation of glutathione in phase II detoxification reactions involving a wide range of xenobiotics, including chemotherapeutic antimetabolites [56]. The *GSTP1* rs1695 polymorphism is defined by a guanine-to-adenine change at position 313, leading to the substitution of isoleucine with valine at codon 105 of the exon 5, resulting in reduced enzymatic activity [56,57]. The evidence regarding the association of this polymorphism with HDMXT-related toxicity is limited and presents inconsistent results [29,57,58]. Our study revealed that carriers of the *GSTP1* rs1695 AA genotype (AA vs. G) exhibited an elevated risk of hematologic toxicity (grades I–IV) (*p* = 0.02). A single study on the association between the *GSTP1* rs1695 polymorphism and MTX toxicity was identified. This study, conducted by Kishi and collaborators in 2017, involved a population of 240 American children of Sub-Saharan African, European, Native American, and East Asian ancestry. The study reported that the *GSTP1* rs1695 GG genotype (GG vs. A) was associated with an elevated risk of neurological toxicity during the continuation phase of leukemia therapy (*p* = 0.006; OR = 2.70; 95%CI = 1.18–6.25) [59]. A meta-analysis by Kim et al. in 2022, of 10 studies in a mixed population (Japan, Brazil, Italy, China, Korea, and France) investigated the role of *GSTP1* rs1695 on platinum-based chemotherapy in adult patients with solid neoplasms (esophagus, lung, and colon). The study found a protective effect of the *GSTP1* rs1695 G allele (G vs. AA) against gastrointestinal toxicity (OR = 0.56; 95%CI = 0.34–0.92), which is consistent with our findings on hematologic toxicity. Furthermore, the authors suggest that the expression of this polymorphism is not uniformly distributed among body tissues, which may elucidate its divergent implications in the development of various types of toxicities [57]. In contrast, the meta-analysis by Gong et al. published in 2021, that included 1922 patients from 13 studies in a mixed population cohort (Japan, China, North America, Bangladesh, Korea, Italy, Egypt, Indonesia, and Myanmar) found an association between the *GSTP1* rs1695 AG/GG genotypes (G vs. AA) and a higher risk of gastrointestinal toxicity (RR= 1.61; 95%CI = 1.18–2.19; *p* = 0.003) and infection (RR = 1.57; 95%CI = 1.00–2.48; *p* = 0.05) in patients with cancer and autoimmune diseases undergoing cyclophosphamide treatment. However, when the study population was stratified in terms of diagnosis, the association between the polymorphism and toxicity was only observed in patients with lupus erythematosus and lupus-related nephrotic syndrome [60].

The conflicting findings regarding the role of polymorphisms in genes involved in MTX pharmacokinetics and pharmacodynamics, as well as the different toxicities observed in patients, are likely due to several factors. These include differences in the study design, population heterogeneity, variations in treatment protocols, small sample sizes, and the necessity for dose adjustments in response to toxicity. Such disparities make it challenging to draw consistent conclusions across studies.

The limitations of our study include the potential influence of concomitant medication that can exacerbate the toxic effects of MTX and the presence of additional genetic variations on the observed toxicities, such as thymidylate synthase (TYMS), which despite its essential function in MTX metabolism, was not chosen for this investigation due to its low prevalence in the examined population and the difficulties associated with procuring a probe for PCR, which includes an insertion/deletion. Additionally, the risk of bias due to the sample size and the heterogeneous nature of the study population in terms of diagnoses and treatment protocols cannot be ruled out. However, our results provide substantial evidence regarding the impact of SNPs on HDMTX-related toxicity in childhood cancer, thereby supporting future research aimed at predicting and mitigating this toxicity. Due to the variability of genetic polymorphisms frequencies among populations, the relationships identified in Spanish patients should be investigated in other populations with distinct genetic ancestries.

Future initiatives to establish a methotrexate (MTX) pharmacogenetic guideline should prioritize the validation of more studies and with greater levels of evidence polymorphisms in PharmGKB, including those in *SLC19A1, SLCO1B1, MTHFR, TYMS, DHFR*, *ABCB1* and *ABCC2*, via extensive and diverse clinical investigations that correlate genotypes with MTX efficacy and toxicity. Explicit guidelines necessitate rigorous clinical validation, prospective research, and the creation of clinical decision-making instruments. Progress in genotyping, bioinformatics, and electronic health record integration is also crucial. Ultimately, success relies on international cooperation and the education of healthcare providers to facilitate implementation and guarantee equal access to tailored MTX therapy.

## 5. Conclusions

HDMTX is an important agent in the treatment of childhood cancer, significantly contributing to improved treatment outcomes and quality of life for affected children. Nevertheless, further investigation is required to elucidate biomarkers that predict its toxic effects. Our study findings indicate that individuals with the AA genotype of *MTHFD1* rs2236225 (*p* = 0.03) and who are of the male sex (*p* = 0.03) are at an elevated risk of developing severe gastrointestinal toxicity. Additionally, our results suggest that hematological toxicity (grades I–IV) is associated with the A allele of *MTHFR* rs1801133 (*p* < 0.01) and the AA genotype of *GSTP1* rs1695 (*p* = 0.02).

## Figures and Tables

**Table 1:** Sociodemographic and clinical variables of 107 patients treated with HDMTX.

Sociodemographic and Clinical Variables	Sex						*p*-Value
	Female 49 (45.79%)			Male 58 (54.21%)			
	*n*	%	Median (p25–p75)	*n*	%	Median (p25–p75)	
**Family history of cancer**	21	42.86		27	46.55		0.70
**Cancer diagnosis**							
*Acute lymphoblastic leukemia*	32	65.31		34	58.62		0.53
*Non-Hodgkin’s lymphoma*	7	14.29		12	20.69		
*Osteosarcoma*	7	14.29		10	17.24		
*Ependymoma*	3	6.12		1	1.72		
*Undifferentiated sarcoma of small cells*	0	0.00		1	1.72		
**Age at diagnosis**			6 (4–8)			6.9 (3–11.5)	0.24
Concomitantly administered antineoplastic drug *	37	75.51		45	77.59		0.80
**Elimination > 48 h**	36	73.47		36	62.07		021
**General toxicity grade I–IV**	33	67.35		50	86.21		**0.02**
**General toxicity grade III–IV**	30	61.22		42	72.41		0.23
**Hematologic toxicity**							
*Hematologic toxicity grade I–IV*	26	53.06		40	68.97		0.09
*Neutropenia grade I–IV*	25	51.02		39	67.24		0.09
*Thrombocytopenia grade I–IV*	12	24.49		27	46.55		**0.02**
*Hematologic toxicity grade III–IV*	22	44.9		33	56.90		0.21
**Gastrointestinal toxicity**							
*Gastrointestinal toxicity grade I–IV*	26	53.6		39	67.24		0.13
*Diarrhea grade I–IV*	6	12.24		9	15.52		0.62
*Mucositis grade I–IV*	7	14.29		19	32.76		**0.03**
*Nausea and vomiting grade I–IV*	20	40.82		25	43.10		0.81
*Gastrointestinal toxicity grade III–IV*	4	8.16		15	25.86		**0.02**
**Liver toxicity**							
*Liver toxicity grade I–VI*	18	36.73		20	34.48		0.80
*Liver toxicity grade III–IV*	14	28.57		13	22.41		0.33
**Kidney toxicity**							
*Kidney toxicity grade I–VI*	2	4.08		5	8.62		0.34
*Kidney toxicity grade III–IV*	2	4.08		2	3.45		0.86
**Pulmonary toxicity**	4	8.16		4	6.90		0.80
**Skin toxicity**	1	2.04		8	12.07		0.14

Qualitative variables: frequency (percentage, %); quantitative variables: non-normal distribution median (p25–p75); *n*: number of patients; * concomitant drugs may include: 6-mercaptopurine, anthracyclines, asparaginase, cyclophosphamide, or vincristine.

**Table 2:** Sociodemographic/clinical variables and toxicity.

Sociodemographic and Clinical Variables	Toxicity	OR	95%CI	*p*-Value
Male sex	General toxicity (grades I–IV)	2.30	1.06–9.07	0.02
	Gastrointestinal toxicity (grades III–IV)	3.87	1.11–17.32	0.02
	Mucositis (grades I–IV)	2.89	1.02–9.07	0.03
	Thrombocytopenia (grades I–IV)	2.66	1.09–6.78	0.02
Diagnosis of ALL	Hepatic toxicity (grades III–IV)	5.81	1.07–108.36	<0.01
	MTX clearance > 48 h	3.16	1.11–9.34	0.02
Diagnosis of NHL	Gastrointestinal toxicity (grades III–IV)	3.93	1.20–12.85	0.03
	Mucositis (grades I–IV)	5.60	1.90–17.37	<0.01
Diagnosis of OS	Gastrointestinal toxicity (grades I–IV)	7.96	2.03–53.13	<0.01
	Nausea and vomiting (grades I–IV)	10.00	2.89–48.86	<0.01
	Hepatic toxicity (grades III–IV)	3.11	1.23–6.11	<0.01
	Clearance MTX > 48 h	6.41	1.49–35.20	0.02
Age at diagnosis	Gastrointestinal toxicity (grades I–IV)	1.71	1.05–1.31	<0.01
	Nausea and vomiting (grades I–IV)	1.20	1.09–1.34	<0.01
Concomitant antineoplastic agent *	Hematologic toxicity (grades III–IV)	2.96	1.06–8.91	0.02

CI: confidence interval; OR: odds ratio; * concomitant drugs may include: 6-mercaptopurine, anthracyclines, asparaginase, cyclophosphamide, or vincristine.

**Table 3:** Multivariate regression analysis of toxicity.

Variables	*q*-Value	*p*-Value	OR	95%CI
**Gastrointestinal toxicity (grades III–IV)**				
*MTHFD1* rs2236225 (AA vs. AG/GG)	0.06	0.03	6.15	1.29–35.41
Sex (male vs. female)	0.06	0.03	3.71	1.20–14.10
**Hematological toxicity (grades I–IV)**				
*MTHFR* rs1801133 (A vs. GG)	0.02	<0.01	4.23	1.73–10.98
*GSTP1* rs1695 (AA vs. G)	0.02	0.02	2.88	1.23–7.17

CI: confidence interval; OR: odds ratio.

## Data Availability

The original contributions presented in this study are included in the article. Further inquiries can be directed to the corresponding author(s).

## Supplementary Materials

The following supporting information can be downloaded at: https://www.mdpi.com/article/10.3390/pharmaceutics17050585/s1. Table S1: *Hardy-Weinberg equilibrium* for the SNPs included in the study; Table S2: Linkage disequilibrium of the studied SNPs.; Table S3: Linkage disequilibrium of the studied SNPs; Table S4: Significant results bivariate analysis.

## Abbreviations

ABC, ATP-binding cassette; ALL, acute lymphoblastic leukemia; ABCB1, ATP-binding cassette subfamily B member 1; ABCC1, ATP-binding cassette subfamily C member 1; ABCC2, ATP-binding cassette subfamily C member 2; CPIC, Clinical Pharmacogenetics Implementation Consortium; dbSNP, Single Nucleotide Polymorphism Database; DHFR, dihydrofolate reductase¸ DNA, deoxyribonucleic acid; GSTP1, glutathione S-transferase pi 1; HDMTX, high-dose methotrexate; HWE, Hardy–Weinberg equilibrium; ID: identification; LD, linkage disequilibrium; MAF, minor allele frequency; MTHFD1, methylenetetrahydrofolate dehydrogenase; MTHFR, methylenetetrahydrofolate reductase; MTR, methionine synthase; MTRR, methionine synthase reductase; MTX, methotrexate; MTXPG, methotrexate polyglutamate; NHL, non-Hodgkin’s lymphoma; NUDT15, Nudix hydrolase 15; PharmGKB, Pharmacogenomics Knowledge Base; PCR, polymerase chain reaction; r^2^, squared correlation coefficient; RNA, ribonucleic acid; SLC, solute carrier family; SLCO1B1, solute carrier organic anion transporter family member 1B1; SNP, single nucleotide polymorphisms; TPMT, thiopurine S-methyltransferase.
