# Supplementary material for: Impact of Pharmacogenetics on High-Dose Methotrexate Toxicity in Pediatric Oncology
Source: Pharmaceutics. 2025 Apr 29;17(5):585. doi: 10.3390/pharmaceutics17050585 (PMC12115323; doi:10.3390/pharmaceutics17050585)
Supplement: Supplementary file 1 [file pharmaceutics-17-00585-s001.zip › pharmaceutics-3533669-supplementary.pdf]

**Table S1.** *Hardy-Weinberg equilibrium* for the SNPs included in the study.

| Chr | SNP | Gene | Minor Allele | Major Allele | Genotype counts | Observed heterozygosity | Expected heterozygosity | p-value |
|---|---|---|---|---|---|---|---|---|
| 1 | rs1801133 | *MTHFR* | A | G | 19/56/32 | 0.5234 | 0.4926 | 0.5624 |
| 1 | rs1801131 | *MTHFR* | G | T | 8/42/57 | 0.3925 | 0.3951 | 1 |
| 1 | rs1805087 | *MTR* | G | A | 23/45/39 | 0.4206 | 0.4888 | 0.1664 |
| 5 | rs1801394 | *MTRR* | G | A | 22/63/22 | 0.5888 | 0.5 | 0.0839 |
| 5 | rs70991108 | *DHFR* | D | I | 20/43/44 | 0.4019 | 0.4748 | 0.1082 |
| 7 | rs1128503 | *ABCB1* | A | G | 23/46/38 | 0.4299 | 0.4902 | 0.2361 |
| 10 | rs717620 | *ABCC2* | T | C | 9/40/58 | 0.3738 | 0.3951 | 0.6244 |
| 11 | rs1695 | *GSPT1* | G | A | 9/48/50 | 0.4486 | 0.4266 | 0.6577 |
| 12 | rs11045879 | *SLCO1B1* | C | T | 1/32/74 | 0.2991 | 0.2673 | 0.4626 |
| 12 | rs4149081 | *SLCO1B1* | A | G | 1/32/74 | 0.2991 | 0.2673 | 0.4626 |
| 14 | rs2236225 | *MTHFD1* | A | G | 15/59/33 | 0.5514 | 0.4859 | 0.2317 |
| 16 | rs246240 | *ABCC1* | G | A | 0/21/86 | 0.1963 | 0.177 | 0.5931 |
| 20 | rs7270101 | *ITPA* | C | A | 2/35/70 | 0.3271 | 0.2981 | 0.5156 |
| 22 | rs5760410 | *ADORA2A* | G | A | 23/44/40 | 0.4112 | 0.4874 | 0.1137 |
| | *Chr: Chromosome; SNP: single nucleotide polymorphism* | | | | | | | |

**Table S2.** Linkage disequilibrium of the studied SNPs.

| Chr | BP | SNP | Chr | BP | SNP | $R^2$ | D' |
|---|---|---|---|---|---|---|---|
| 12 | 21229685 | rs11045879 | 12 | 21225087 | rs4149081 | 0.847547 | 0.929313 |

*Chr: Chromosome; BP: Physical position (base-pair); SNP:single nucleotide polymorphism.*

**Table S3.** Minor allele frequencies for the SNPs studied.

| Chr | SNP | Gene | Minor Allele | Major Alelle | MAF |
|---|---|---|---|---|---|
| 1 | rs1801133 | *MTHFR* | A | G | 0.4393 |
| 1 | rs1801131 | *MTHFR* | G | T | 0.2710 |
| 1 | rs1805087 | *MTR* | G | A | 0.4252 |
| 5 | rs1801394 | *MTRR* | G | A | 0.5000 |
| 5 | rs70991108 | *DHFR* | D | I | 0.3879 |
| 7 | rs1128503 | *ABCB1* | A | G | 0.4299 |
| 10 | rs717620 | *ABCC2* | T | C | 0.2710 |
| 11 | rs1695 | *GSPT1* | G | A | 0.3084 |
| 12 | rs11045879 | *SLCO1B1* | C | T | 0.1589 |
| 12 | rs4149081 | *SLCO1B1* | A | G | 0.1589 |
| 14 | rs2236225 | *MTHFD1* | A | G | 0.4159 |
| 16 | rs246240 | *ABCC1* | G | A | 0.0981 |
| 20 | rs7270101 | *ITPA* | C | A | 0.1822 |
| 22 | rs5760410 | *ADORA2A* | G | A | 0.4206 |

Chr: chromosome; SNP: single nucleotide polymorphism; MAF: minor allele frequency.

**Table S4.** Significant results bivariate analysis

| | Genotype | N | No N(%) | Yes N(%) | Rf** | OR | 95%CI | p-valor |
|---|---|---|---|---|---|---|---|---|
| ***GSPT1* rs1695** | | | | | | | | |
| **Overall toxicity grade III-IV** | AA | 50 | 11(22.0) | 39(78.0) | AG | 2.79 | 1.22-6.59 | **0.04*** |
| | AG | 48 | 21(43.8) | 27(56.2) | | | | |
| | GG | 9 | 3(33.3) | 6(66.7) | | | | |
| | A- | 98 | 32(32.7) | 66(67.3) | | | | |
| | G- | 57 | 24(42.1) | 33(57.9) | G- | 2.55 | 1.02-6.70 | **0.03*** |
| **Hematological toxicity Neutropenia grade I-IV** | AA | 50 | 14(28.0) | 36(72.0) | AG | 2.79 | 1.22-6.59 | **0.04*** |
| | AG | 48 | 25(52.1) | 23(47.9) | | | | |
| | GG | 9 | 4(44.4) | 5(55.6) | | | | |
| | A- | 98 | 39(39.8) | 59(60.2) | | | | |
| | G- | 57 | 29(50.9) | 28(49.1) | G- | 2.64 | 1.11-6.50 | **0.02** |
| ***MTHFR* rs1801133** | | | | | | | | |
| **Overall gastrointestinal toxicity grade III-IV** | AA | 19 | 19(100.0) | 0(0.0) | AA | | - | **0.04*** |
| | AG | 56 | 45(80.4) | 11(19.6) | | $2.82^{e+07}$ | $6.85^{e-43}$ - NA | |
| | GG | 32 | 24(75.0) | 8(25.0) | | $3.85^{e+07}$ | $1.25^{e-49}$ - NA | |
| | A- | 75 | 64(85.3) | 11(14.7) | | | | |
| | G- | 88 | 69(78.4) | 19(21.6) | AA | Inf | 1.14-Inf | **0.02*** |

Table S4 (continuación)

| | Genotype | N | | | Rf** | OR | 95%CI | p-valor |
|---|---|---|---|---|---|---|---|---|
| | | | ***MTHFR* rs1801133** | | | | | |
| | | | **No N(%)** | **Yes N(%)** | | | | |
| **Overall Hematological toxicity grade I-IV** | AA | 19 | 7(36.8) | 12(63.2) | GG | 3.99 | | **0.01** |
| | AG | 56 | 15(26.8) | 41(73.2) | | | 1.62-10.28 | |
| | GG | 32 | 19(59.4) | 13(40.6) | | | - | |
| | A- | 75 | 22(29.3) | 53(70.7) | GG | 3.45 | 1.36-9.16 | **<0.01** |
| | G- | 88 | 34(38.6) | 54(61.4) | | | | |
| **Neutropenia grade I-IV** | AA | 19 | 8(42.1) | 11(57.9) | | | | |
| | AG | 56 | 16(28.6) | 40(71.4) | GG | 3.65 | 1.49-9.32 | **0.02** |
| | GG | 32 | 19(59.4) | 13(40.6) | | | - | |
| | A- | 75 | 24(32.0) | 51(68.0) | GG | 3.07 | 1.21-8.02 | **0.01** |
| | G- | 88 | 35(39.8) | 53(60.2) | | | | |
| | | | ***MTHFD1* rs2236225** | | | | | |
| | | | **I-II N(%)** | **III-IV N(%)** | | | | |
| **Overall gastrointestinal toxicity grade I-II-/III-IV** | AA | 9 | 3(33.3) | 6(66.7) | | | 2.09-91.08 | |
| | AG | 35 | 25(71.4) | 10(28.6) | GG | 12.00 | | **0.01*** |
| | GG | 21 | 18(85.7) | 3(14.3) | | | | |
| | A- | 44 | 28(63.6) | 16(36.4) | | | | |
| | G- | 56 | 43(76.8) | 13(23.5) | G- | 6.38 | 1.17-45.00 | **0.01*** |

Table S4 (continuación)

| | Genotype | N | | | Rf** | OR | 95%CI | p-valor |
|---|---|---|---|---|---|---|---|---|
| ***ABCB1 rs1128503*** | | | | | | | | |
| | | | **I-II N(%)** | **III-IV N(%)** | | | | |
| **Overall gastrointestinal toxicity grade I-II-/III-IV** | AA | 14 | 13(92.9) | 1(8.3) | | | | |
| | AG | 28 | 18(64.3) | 10(35.7) | - | | | |
| | GG | 23 | 15(65.2) | 8(34.8) | | | | |
| | A- | 42 | 31(73.8) | 11(26.2) | | | | |
| | G- | 51 | 33(64.7) | 18(35.3) | AA | 6.93 | 0.90-317.32 | **0.049** |
| ***DHFR* rs70991108** | | | | | | | | |
| | | | **I-II N(%)** | **III-IV N(%)** | | | | |
| **Gastrointestinal toxicity-nausea and vomiting grade I-IV** | DD | 20 | 12(60.0) | 8(40.0) | | | | |
| | DI | 43 | 32(74.4) | 11(25.6) | | | | |
| | II | 44 | 18(40.9) | 26(59.1) | | | | |
| | D- | 63 | 44(69.8) | 19(30.2) | D- | 3.30 | 1.38-8.11 | **<0.01** |
| | I- | 87 | 50(57.5) | 37(42.5) | | | | |
| ***MTR* rs1805087** | | | | | | | | |
| | | | **No N(%)** | **Yes N(%)** | | | | |
| **Neutropenia + thrombocytopenia grade I-IV** | AA | 39 | 19(48.7) | 20(51.3) | | | | |
| | AG | 45 | 32(71.1) | 13(28.9) | | | | |
| | GG | 23 | 17(73.9) | 6(26.1) | | | | |
| | A- | 84 | 51(60.7) | 33(39.3) | | | | |
| | G- | 68 | 49(72.1) | 19(27.9) | G- | 2.69 | 1.10-6.68 | **0.01** |

*p-value for the Fisher's test; ** Rf- reference category; Inf- Infinite.
